# Distributed expertise: qualitative study of a British network of multidisciplinary teams supporting parents of children with chronic kidney disease

**DOI:** 10.1111/cch.12141

**Published:** 2014-05-14

**Authors:** V Swallow, T Smith, N J A Webb, L Wirz, L Qizalbash, E Brennan, A Birch, M D Sinha, L Krischock, J van der Voort, D King, H Lambert, D V Milford, L Crowther, M Saleem, A Lunn, J Williams

**Affiliations:** * School of Nursing, Midwifery and Social Work, Faculty of Medical and Human Sciences, Manchester Academic Health Sciences Centre, University of ManchesterManchester, UK; † Royal Manchester Children's HospitalManchester, UK; ‡ Health Psychology (Old Ward 1 Offices), Royal Victoria InfirmaryNewcastle, UK; § The Great North Children's Hospital, Newcastle upon Tyne Hospitals NHS Foundation TrustNewcastle, UK; ¶ Great Ormond Street Children's HospitalLondon, UK; ** Nephrology, Alder Hey Children's NHS Foundation TrustLiverpool, UK; †† Department of Pediatric Nephrology, Evelina Children's Hospital, Guy's & St Thomas' NHS Foundation TrustLondon, UK; ‡‡ Sydney Children's HospitalRandwick, NSW, Australia; §§ Children's Kidney Centre, UHWCardiff, UK; ¶¶ Yorkhill Children's HospitalGlasgow, UK; *** Birmingham Children's HospitalBirmingham, UK; ††† Royal Manchester Children's Hospital, Central Manchester University Hospitals NHS Foundation TrustManchester, UK; ‡‡‡ University of Bristol Children's Renal Unit, Bristol Royal Hospital for ChildrenBristol, UK; §§§ Children's Renal and Urology Unit, Nottingham Children's Hospital, Nottingham University Hospitals NHS Trust, QMC CampusNottingham, UK; ¶¶¶ School of Environment, Education and Development, University of ManchesterUK

**Keywords:** children, CKD, distributed expertise, long-term conditions, multidisciplinary teams, parents

## Abstract

**Background:**

Long-term childhood conditions are often managed by hospital-based multidisciplinary teams (MDTs) of professionals with discipline specific expertise of a condition, in partnership with parents. However, little evidence exists on professional–parent interactions in this context. An exploration of professionals' accounts of the way they individually and collectively teach parents to manage their child's clinical care at home is, therefore, important for meeting parents' needs, informing policy and educating novice professionals. Using chronic kidney disease as an exemplar this paper reports on one aspect of a study of interactions between professionals and parents in a network of 12 children's kidney units in Britain.

**Methods:**

We conducted semi-structured, qualitative interviews with a convenience sample of 112 professionals (clinical-psychologists, dietitians, doctors, nurses, pharmacists, play-workers, therapists and social workers), exploring accounts of their parent-educative activity. We analysed data using framework and the concept of distributed expertise.

**Results:**

Four themes emerged that related to the way expertise was distributed within and across teams: (i) recognizing each other's' expertise, (ii) sharing expertise within the MDT, (iii) language interpretation, and (iv) acting as brokers. Two different professional identifications were also seen to co-exist within MDTs, with participants using the term ‘we’ both as the intra-professional ‘we’ (relating to the professional identity) when describing expertise within a disciplinary group (for example: ‘As dietitians we aim to give tailored advice to optimize children's growth’), and the inter-professional ‘we’ (a ‘team-identification’), when discussing expertise within the team (for example: ‘We work as a team and make sure we're all happy with every aspect of their training before they go home’).

**Conclusions:**

This study highlights the dual identifications implicit in ‘being professional’ in this context (to the team and to one's profession) as well as the unique role that each member of a team contributes to children's care. Our methodology and results have the potential to be transferred to teams managing other conditions.

## Introduction

Long-term childhood conditions are often managed by national networks of hospital-based multidisciplinary teams (MDTs) comprising professionals with expertise in the care of children with a specific condition such as chronic kidney disease (CKD). The UK National Service Framework for Renal Services (DOH 2006) acknowledges that a diagnosis of CKD can be devastating for the whole family. MDTs therefore, aim to work in partnership with parents who often deliver most of the clinical care at home using care-plans that are age and culturally appropriate (Scott *et al*. 1997; DOH 2006). Parent-support by a MDT that offers its services from the point of diagnosis and puts an emphasis on preventive care may help to achieve, maintain and improve physical and mental health and social functioning in patients and parents (BRS 2002; Menon *et al*. 2009; Ajarmeh *et al*. 2012).

The paediatric nephrology network in Britain (England, Scotland and Wales) comprises 12 children's kidney units, each with a dedicated MDT. The MDT usually becomes the focus for families to seek specialist support, advice and guidance on clinical care, emotional, social and educational issues (DOH 2006). The MDTs aim to meet parents' needs as part of an integrated, co-ordinated model of service provision within a complex network of primary, secondary and tertiary healthcare systems. A number of challenges for MDTs are identified (BRS 2002; DOH 2006) with guidance advocating that professionals are appropriately trained to support this role.

The benefits of MDT care for children with CKD, over other models, have been shown to include better: anaemia management, bone mineral metabolism, nutrition, renal disease progression and preparation for dialysis (Menon *et al*. 2009; Ajarmeh *et al*. 2012). However, little evidence exists about the way MDTs function or of how MDT members can effectively share their discipline-specific knowledge with parents and each other. Using CKD as an exemplar, but also acknowledging the non-categorical nature (Stein & Jessop 1982) of some features of MDT support for parents, the research described here is one aspect of a study of social interaction in the network of 12 children's kidney units in Britain. The wider study involves: (Phase 1) a survey mapping MDTs' parent-educative activities (Table 1 summarizes the results), (Phase 2) exploration of MDTs' accounts of their parent-teaching activity, and (Phase 3) ethnographic case-studies of professional/parent interactions during shared-care in two of the units.

**Table 1:** Summary of number of staff reported in each category in the respective units

Staff category	Units												Total
	1	2	3	4	5	6	7	8	9	10	11	12	
Paediatric nephrologist	3 T&R	5 T&R	6 R	5 T&R	6 T&R	7 T&R	7 R	4 T&R	5 T&R	5 R	4 T&R	7 R	64
Junior doctor	3 T&R	4 T&R	4 T&R	2 T&R	2 T&R	7 T&R	7 No	5 T&R	1 T&R	4 R	4 R	4 R	47
Renal specialist nurse	1.2 T&R	2 T&R	6 T&R	5 T&R	2 T&R	4 T&R	10 T&R	3 T&R	6 T&R	5 T&R	2 T&R	2 T&R	48.2
Ward nurse	14 T&R	22 T&R	22 T&R	9 T&R	21 T&R	44 T&R	40 T&R	20 T&R	36 T&R	21 T&R	36 R	38 T&R	323
Haemodialysis nurse		9 T&R	12 T&R	5 T&R	20 T&R	23 T&R	19 T&R	2.5 T&R	8 T&R	14 T&R	7 R	10 T&R	129.5
Peritoneal nurse		18 T&R	25 T&R	14 T&R	20 T&R	30 T&R	30 T&R	10 T&R	19 T&R	24 T&R	25 R	30 T&R	245
Healthcare assistant or clinical support worker	3 T&R	3 No	4 T&R	4 No	3 T	2 No	4 T&R	4 No	3 R	3 T	2 R	4 No	39
Dietician	1 T&R	2 T&R	2 T&R	1 T&R	2 T&R	1 T&R	1 T&R	2 T&R	2 T&R	2 T&R	1 T&R	2 T&R	19
Counsellor or therapist	1 T&R	0	0	0	0	1 No	1 R	0	0	0	0	0	3
Clinical psychologist	1 T&R	No	2 R	0.6 Paed	2 R	1 T&R	0	0	1	1 T&R	1 R	1	10.6
Pharmacist	1 T&R	1 T	1	1 No	1 T&R	1 T&R	1 No	1 T&R	1 No	1	1 R	1 T&R	12
Play-specialist	1 T&R	1 T	2 T&R	1 T&R	1 T&R	2 T&R	2 T&R	1 T&R	1 T&R	1 T&R	2 T&R	1 T&R	16
Social worker	1 No	1 T	1 R	1 R	1 No	1 T&R	1 R	1 T&R	1 T&R	0	0	0	9

T = teaches parents; R = reinforces others' parent-teaching; No = neither teaching or reinforcing others' teaching; T&R = teaches parents and reinforces others' teaching; Paed = paediatric.

This article describes the analysis of data obtained during qualitative interviews with MDT professionals in Phase 2. We were particularly interested in exploring participants' accounts of their parent-educative activities within the context of both a MDT and a national network, so a key concept used in our analysis was ‘distributed expertise’. This approach requires professionals to negotiate and work at high-trust relationships with other professionals (Edwards 2004; Edwards *et al*. 2009). When considering the issue of inter-professional collaborations in the MDT context, distributed expertise is viewed as a collective competence spread across systems that is drawn upon to accomplish specific tasks. The idea of distributed professional expertise calls for a new version of professional competence and identity which is resourceful, outward-looking and enables individual professionals to recognize what others can offer and what they themselves can offer within the team. When tasks and activities are performed in a team environment, the co-ordination of resources is critical to effective performance. Furthermore, MDTs are widely seen as teams of skilled professionals who work together for the common goal of patient care (Engestrom & Middleton 1996; Polit & Beck 2010). However, professionals from different disciplines have distinct areas of professional expertise and task responsibility, so the work of the team depends on a combination of the effectiveness of professionals' distinct competences, and crucially, the effective co-ordination of the MDT around the needs of patients and families.

In summary, the way professionals share clinical expertise with parents is not currently well understood so research is needed which provides a better understanding of professionals' views on the way they share discipline-specific clinical expertise with parents. The data reported here address this gap in line with our published protocol (Swallow *et al*. 2012).

## Methods

To obtain in-depth understanding of professionals' accounts we used an interpretative approach involving qualitative methods (Green & Thorogood 2014). A total of 112 health professionals (Table 2) from the 12 units participated in 13 group and seven individual, semi-structured interviews lasting on average 50 minutes and structured by a topic guide. Discussion focussed on professionals' parent-educative activity and built on the literature, including the parent-teaching activities we previously described: assessing learning needs, creating learning opportunities, implementing teaching strategies, acting as interpreters and ambassadors and assessing learning progress (Knafl & Gilliss 2002; Tong *et al*. 2008; Swallow *et al*. 2009; Parker *et al*. 2013). Group interviews involved professionals who were available within the study timeframe. Each group interview comprised a combination of disciplines but for logistical reasons no entire team was able to participate in any one group. Individual interviews were offered to those who were interested but not available for group interviews. Interviews were digitally recorded and transcribed verbatim.

**Table 2:** Numbers of participants across the 12 units from each discipline

Discipline	Number participating
Clinical psychologists	7
Doctors	Consultant paediatric nephrologists: 28 Registrars: 2
Dietitians	9
Nurses†	48
Pharmacists	3
Play workers	7
Social workers	6
Therapists	2

Included the roles of specialist nurse, nurse consultant, nurse specialist, clinical nurse specialist, associate nurse specialist, advanced nurse practitioner, staff nurse, senior staff nurse, junior sister, sister, matron, ward manager, research nurse, clinic nurse, community nurse, ward nurse, nurse working in haemodialysis, peritoneal dialysis or transplant.

### Data analysis

Data were analysed using Framework which is systematic, rigorous and grounded in the data. Transcripts are analysed through five iterative stages: (1) familiarization with the data; (2) identification of a framework; (3) indexing; (4) charting; and (5) mapping/interpretation (Ritchie & Lewis 2003). Initially, two researchers independently read/coded the first transcript, searching for patterns in the data, mapping connections and seeking explanations for patterns before comparing and discussing these. A data sample was coded independently by a third researcher and then all three discussed it until a consensus was reached. The framework was then applied (3) to all transcripts. Each coded transcript was then (4) transferred to a spread-sheet for charting. Stages (4) and (5) were facilitated by coding data from disciplinary datasets across and between the 12 units to ensure inter-rater agreement on coding and data saturation. Emerging themes supplemented interview topics. The iterative process involved moving back and forwards between stages (Swallow *et al*. 2003) thus enabling interpretation and rearrangement of data for more detailed analysis/interpretation, and helping us identify further lines of enquiry to pursue (Ritchie & Lewis 2003).

### Ethical considerations and rigour

We received approval from an NHS research ethics committee (reference: 09/H1002/92) and each participating Trust's Research and Development Department. Participants were assured that data would be stored in a secure place and anonymized before reporting. Written and verbal study information was provided and written consent obtained. Data were collected by researchers previously unknown to most participants.

## Findings

In our data, two different professional identities were seen to co-exist within MDTs. Professionals frequently used the term ‘we’ as the intra-professional ‘we’ (e.g. ‘As dietitians we aim to give tailored advice to optimize children's growth’), and as the inter-professional, team-centred ‘we’ when discussing expertise within the team (e.g. ‘We work as a team and make sure we're all happy with every aspect of their training before they go home’). Four themes emerged that related to the way expertise was distributed within and across teams:

### Recognizing each other's' expertise

The various professionals offered different kinds of expertise to the co-management of children. This distributed expertise explains the group's capacity (rather than just each individual's) to learn, act on and transform the problems of practice. One participant explained how this distributed expertise has evolved over time:

… there was [initially] myself and one renal nurse … over the years we recognised additional [staff] appointments we needed and for most of those we ended up making bids for charitable funding. So … we felt we needed a renal dietitian, … then the social worker … and psychologist post came that way … Individual units [in Britain] have been doing that, over the years. (Doctor_96)

The actions and approaches of each professional were believed to impact on parents' experiences of caring for their child. MDTs differed in the way they were configured, but without prompting, interviewees frequently discussed in detail the way team members work collaboratively and what their personal team roles involved. There was a sense that team members often supported each other as well as the families, for example a clinical psychologist explained:

My role in the team is [also] to help everybody [colleagues] think psychologically about what's happening … I think our team works very well together in that we each have input for families for various reasons at different times. (Clinical Psychologist_15)

Although team cohesion was pivotal in supporting parents' learning, role differentiation was also central to the way participants described their roles. As if to emphasize this, there were many unsolicited accounts from participants who stressed the important role of other disciplines within their team, as well as their own, as the following data excerpt illustrates:

We all have a role … the nurses get parents ready for home, you know, NG [naso-gastric] feeding first-up, can the parents be told how to do it? … we work as a team and make sure we're all happy with every aspect of training before they go home, so they're safe to either be giving the medicines, certain feeds, knowing when to call for help. (Doctor_7)

The MDT also functioned to reinforce information, as defined in Phase 1 (Table 1), consequently parents may receive the same piece of information from different professionals at different times, or one professional may reinforce some information they know a member of another discipline had provided to parents. This division of labour coupled with a tacit understanding that each member would ‘back-up’ the other was often reported as fundamental to the way MDTs operate.

Doctors and nurses often acknowledged the important practical role of social workers, play-workers, clinical psychologists and therapists, for example:

… with the social worker there is a much more practical element [for instance] they realise they [parents] are going to be stuck with transport …. sometimes Play-workers can get a different rapport with the child/family, sometimes families just won't open up to you and they would do to the Play-worker. We also support parents to see the value of the Clinical Psychologist or Social Worker. (Nurse_22)

Moreover, play-workers often articulated the value of team-working themselves, as this quotation illustrates:

I go to the MDT for more information … We work quite well as a team. (Play-worker_2)

Some disciplines also played the role of advocate within families:

… sometimes older children don't want to tell their parents everything … if we build a rapport with them when they come in for a while, we find that quite often they'll talk to us about things they're worried about, you know, they don't want to burden their mum. (Play-worker_109)

Pharmacists also highlighted MDT meetings as a forum for professionals to advocate on behalf of parents, for example parents may say something to the pharmacist that suggests they do not fully understand a medication issue, so the pharmacist would discuss this with the team on the parents' behalf.

The respective disciplines were recognized to complement and support each other in supporting families. The following quotation further emphasizes this:

… you need different disciplines in the team … part of our role is to look and say what is causing particular stress [for families]. (Social Worker_30)

This quality of recognition within the MDT was, therefore, seen as important in enabling the different disciplinary competences to be effective.

### Sharing expertise within the MDT

The data contain numerous examples of information sharing within the MDT both in teams managing the condition and in articulating uncertainty about parents' understandings. For example:

So when I first meet [parents] I will go in and say, ‘So what's going on for you and what do you understand of what's going on?’ And that already kind of gets them saying what they've taken in because I'll know probably that they've been told … but it also gives me the understanding of, ‘okay, they haven't understood that’. (Clinical Psychologist_81)

Children with CKD may need input from a range of other specialist teams such as urology, cardiology and surgery, and the renal MDT might advocate for parents with these specialities. The regular MDT meeting was, therefore, an important focus of discussion in many interviews, as one doctor explained:

… we have meetings once a week … The focus is psycho-social … So we're there to link up with the Social Worker, Play-worker … and so on. (Doctor_44)

Another doctor highlighted the benefit of MDT communication on ward rounds or during ‘hand-overs’:

… they [parents] know management plans are discussed that all [MDT members] agree with … if you're new, you're seeing different consultants and your kid's been pretty sick, so you latch onto the first one [consultant] and then think, ‘oh my god, we're going to get a new one, do they know what's happening?’ So, if they know the ‘hand-over’ is a way of dealing with it, they're much, much happier. (Doctor_113)

Teams might also use regular communications to raise concerns about parents' concordance with a child's management plan or to alert colleagues about parents' possible literacy or numeracy problems. Sharing information within the teams was widely reported as an important teaching strategy as well as a way of optimizing the team's understanding of any particular communication needs parents have revealed to individual team members.

### Language interpretation

Teams and individuals highlighted the challenge of communicating complex information to parents whose first language was not English. For example, limited availability of translators was often a concern:

… for the large cities [names two cities] Asians make up 60% of the population and it's [the need for translators is] a big issue, and certainly in terms of resources to address that, we just struggle because it's not there. (Pharmacist_46)

Other participants described the challenge of communicating with parents even when translators were available, for instance:

… the interpreter had placed their own emphasis of importance on what I said … The only way I could sense it was happening was because of the non-verbals. (Doctor_49)

Working with an interpreter was described as an ‘art’ by some participants, as one articulated:

We're [MDTs] discouraged from using family members as interpreters … to be sure it's somebody independent that's relayed that message …. [you need] to make sure that you're focusing on the parent and not the interpreter. (Dietitian_72)

Another challenge related to maintaining confidentiality:

Sometimes the interpreter will be from within the [parents' own] community … it means people in the wider community know the business of the individual. (Nurse_59)

This often linked with a general lack of control about what the translator is saying and on occasions the translator taking a stance as if they are part of the MDT decision-making.

Another communication challenge related to a combination of the ‘protector’ role sometimes adopted by fathers, with the fact that English may not be the parents' first language:

… we make sure that even if we've got a dad that speaks English and a mum that doesn't, we get an interpreter … (we) have had doubts in the past about dads actually translating what we want the mother to know … (Nurse_50)

The fidelity of what interpreters say and their familiarity with clinical language was a concern:

… you give a big whole spiel and then they [interpreter] say, a couple of words and you think, ‘you haven't told them’. (Social Worker_41)

The data also indicate that even parents with English as a first language can struggle to understand some explanations, as a play-worker explained:

Often I sit on home visits [with another discipline], me being a non-medical person, and hear lots of words and think: ‘no one's going to know what that means’ … it is important to remember that all the time and simplify language. (Play-specialist_3)

### Acting as brokers

Regardless of parents' language preference, MDT members frequently drew on their own and each other's' expertise to broker for parents in and out of the hospital setting:

… it's really important to have someone with you when you talk to them [parents], partly because you get two opinions as to how that family are dealing with information. (Doctor_9)

This brokering role could take many forms, including mediating:

I did quite a few phone calls on her [mother's] behalf to the special educational needs co-ordinator in the secondary school. (Clinical Psychologist_16)

A Consultant Nephrologist described informing junior medical staff about the benefits for parents of being able to access the MDT:

I tell them [junior Drs] to take into consideration the psychological situation of the family. (Doctor_14)

For dietitians, brokering can involve teaching the school [on the parents' behalf] about special [renal] diets … meeting with a community nurse, a [school] cook and a class teacher. Some nurses also visit schools:

I've recently been to a local school … reassuring the school and the teachers … I will go back and just check what they [Teachers] are doing and then we can sign off. (Nurse_91)

Social workers, meanwhile, may liaise with parents' employers, or building societies/banks to:

… ask if they [parents] could have a few months respite from paying … organizing disability living allowances for families … it's actually a very good way of getting to know the family … we've written lots of letters, e.g. immigration, or to get a suitable house for a family … (Social Worker_39)

MDT members often act as learning brokers for parents within and outside the team, as well as in the hospital environment and in the community. This brokering role related to practical and theoretical aspects of clinical care-giving and helped professionals to better understand parents' learning needs.

## Discussion and conclusion

We believe this is the first study to elicit first-hand accounts from renal MDT members on their parent-educative activity. Our data illuminate previously un-reported reflections by professionals. Most notably, our findings have the potential to extend understanding of the way professionals individually and collectively promote parents' clinical skill and knowledge development around CKD management. Drawing on the concept of distributed expertise (Edwards *et al*. 2009), helped us capture, through our derived themes ‘*Recognising each other's expertise*’ and ‘*Sharing expertise within the MDT*’, the idea that different professionals offer different kinds of expertise in the co-management of a child's condition. This expertise includes individuals' specialist knowledge and the skills to promote that knowledge within MDTs and with parents. Our data suggest that professional knowledge is not just a stable body of facts that can be acquired through participation in accepted practices, but that it can be reconstructed dynamically as individuals work together.

Distributed expertise also promotes understanding of the range of individually held knowledge bases such as those in our study, and individuals' experience in specific situations, as well as the scope for negotiating the use of expertise in complex situations. Therefore, distributed expertise explains the MDT's capacity (rather than just the individual's) to learn, act on and transform the problems of managing CKD and sharing care with parents.

The concept of ‘identity’ also helps to interpret the significance of professionals' accounts of sharing individual and collective expertise with parents in particular in relation to the themes ‘language interpretation’ and ‘acting as brokers’. Two different identifications were seen to co-exist within MDTs. Professionals frequently used the term ‘we’ when reflecting on their parent teaching activity; the concept of identity helps us interpret this as the intra-professional ‘we’, e.g. ‘As social workers we aim to …’ and the inter-professional ‘we’, e.g. ‘In our team we provide … ’. Wenger suggests that there is a profound connection between identity and practice (Lave & Wenger 1991; Wenger 1998; Lingard *et al*. 2002). The use of the term ‘we’ may negatively affect parents' interpretation of professionals' meanings or lead to confusion about their own identity in shared-caring, as discussed in our earlier research (Swallow 2008). This merits investigation in future research.

In our data there were no examples of implicit disciplinary knowledge being made explicit to other MDT members, this suggests that boundaries around specialisms had already been broken down and that professionals had found a common language that made knowledge accessible to colleagues from other disciplines. However, what is still unclear from the data discussed here is whether that common language is accessible to parents, or if it is, how long it takes for this common language to be understood and/or used by parents.

### Implications for practice, policy, professional education and research

This study is a reminder of the need for effective MDT working, and the unique role that each professional performs. Some professionals may not have a direct role in teaching parents about clinical care, but their skills are crucial in identifying psychosocial factors which need to be considered in planning and implementing parent-education. This could include understanding parents' adjustment and coping styles, learning capacity or mental health issues which could impact on parents' ability to engage with clinical care. An MDT with this enhanced understanding is well placed to take a comprehensive, holistic view of the family's needs, and plan appropriate and effective parent-educative activity. Alongside their role in supporting parents' adjustment, information that has been shared with parents by other disciplines can also be revisited and reviewed enabling any uncertainty by parents to be addressed by the MDT. This has the potential to encourage partnership in decision-making, with an agreed care-plan that supports parents. This is particularly important for implementation of policies which propose that children's health care is family-centred and that parents are supported as active partners in their child's health care (Feveile *et al*. 2007; RCPCH 2006; Shields *et al*. 2012). Our findings can also be integrated into the curricula for professionals' education, and current professionals could use our thematic findings to inform their work of supporting families as part of an MDT.

Most research that focuses on MDT management of CKD has been conducted in North America and has not tended to explore professionals' accounts of the way they individually and collectively support parents. The findings from this study build on this work, but further research to explore professionals' *patient-educative* as well as *parent-educative* strategies, and how to work with interpreters would add to our understanding of the challenges of supporting families living with CKD and other long-term conditions as patients prepare for the transition towards adult services. Such a study could explore facilitators/ barriers to interacting with patients of different ages about their own management.

### Strengths and limitations of the study

Retrospective qualitative interviews generated insights that would not have been available through other methods. We were able to uncover professionals' strategies for supporting parents, hear how they recognize distributed expertise within the team, and understand the way they exploit distributed expertise for the benefit of families. This inductive research allowed new participants to discuss our interpretations of findings from preceding interviews.

Although we recruited a diverse sample from the 12 MDTs and across the disciplines represented, by recruiting a convenience sample we potentially limited the findings to professionals who were prepared to openly discuss their parent-teaching activity, but it would not have been feasible to recruit all MDT members within the project time scale. When reflecting on their interview participation many participants later told the researchers that it was a unique and valuable opportunity to consider the parent-teaching component of their role. However, responses may have been biased by the respondents feeling they needed to please the interviewer so limited recognition of each other's roles within the MDT would not necessarily have been identified. It is also possible that we only uncovered examples of how MDTs work well, without being able to identify any conflicts that might exist within the team. Future research using methodologies, such as individual interviews only or ethnographic/observational methods such as we used in Phase 3 where we reported MDTs and parents negotiating common ground (Swallow *et al*. 2013), could tease out areas where MDTs do not work well in sharing expertise.

The study's condition-specific focus means that while our design could potentially be transferred to other clinical specialities because of the non-categorical nature of some aspects of MDT parent-support, we make no claims to our results being generalizable to other settings. Finally, by respecting participants' confidentiality in this small network of MDTs we do not indicate whether quotations were derived from individual or group interviews. This limits our ability to illustrate the significance of the term ‘we’ in relation to the use of inter- and intraprofessional identifications, future research with larger numbers needs to investigate this.

## Conclusion

There is a dearth of qualitative research on professionals' individual and collective parent-educative activities. Our research explores and discusses the way professionalism works in teams where professionalism requires both a separate identification in which one's discipline can find expression in the interests of the ‘patient and family’, and an awareness of how one's own and others' roles and expertise needs to be both distributed and co-ordinated in the successful action of the team. Further research using different methods would add to our understanding of the challenges of supporting families living with CKD. Our methodology and results have the potential to be transferred to MDTs managing other conditions.

**Key messages**

- A diagnosis of childhood CKD can be devastating for the whole family; it requires extensive communication and support to minimize anxiety and uncertainty, and the renal MDT usually becomes the focus for parents to seek specialist support, advice and guidance when learning to manage their child's CKD at home.
- There is a dearth of qualitative research on the way professionals' individually and collectively provide this specialist support for parents; this study highlights the parent-educative role of professionals in a national nephrology network through first-hand accounts of MDT members.
- Using the concept of distributed expertise in this research helped to illuminate the way expertise is distributed within and across the 12 MDTs in Britain (England, Scotland and Wales)
- In our study 112 professionals (clinical-psychologists, dietitians, doctors, nurses, play-specialists, pharmacists, therapists and social workers) described the way they recognize each other's' expertise, share expertise within the MDT, work with interpreters and act as learning brokers as part of their parent-educative role.
- Engaging the full range of disciplines in research can enable them to articulate their individual and collective parent-educative skills, shape future service developments and inform education of novice practitioners.
